# Molecular pathogenesis and targeted therapeutics in Ewing sarcoma/primitive neuroectodermal tumours

**DOI:** 10.1186/2045-3329-2-6

**Published:** 2012-02-01

**Authors:** Fergal C Kelleher, David M Thomas

**Affiliations:** 1 Department of Medical Oncology, St. Vincent's University Hospital, Dublin, Ireland; 2 Sarcoma Service, Department of Cancer Medicine, Peter MacCallum Cancer Centre, Melbourne, Victoria, Australia

## Abstract

**Background:**

Ewing sarcoma/PNET is managed with treatment paradigms involving combinations of chemotherapy, surgery, and sometimes radiation. Although the 5-year survival rate of non-metastatic disease approaches 70%, those cases that are metastatic and those that recur have 5-year survival rates of less than 20%. Molecularly targeted treatments offer the potential to further improve treatment outcomes.

**Methods:**

A PUBMED search was performed from 1997 to 2011. Published literature that included the topic of the Ewing sarcoma/PNET was also referenced.

**Results:**

Insulin-like growth factor-1 receptor (IGF-1R) antagonists have demonstrated modest single agent efficacy in phase I/II clinical trials in Ewing sarcoma/PNET, but have a strong preclinical rationale. Based on in vitro and animal data, treatment using antisense RNA and cDNA oligonucleotides directed at silencing the EWS-FLI chimera that occurs in most Ewing sarcoma/PNET may have potential therapeutic importance. However drug delivery and degradation problems may limit this therapeutic approach. Protein-protein interactions can be targeted by inhibition of RNA helicase A, which binds to EWS/FLI as part of the transcriptional complex. Tumour necrosis factor related apoptosis inducing ligand induction using interferon has been used in preclinical models. Interferons may be incorporated into future chemotherapeutic treatment paradigms. Histone deacetylase inhibitors can restore TGF-β receptor II allowing TFF-β signalling, which appears to inhibit growth of Ewing sarcoma/PNET cell lines in vitro. Immunotherapy using allogeneic natural killer cells has activity in Ewing sarcoma/PNET cell lines and xenograft models. Finally, cyclin dependent kinase inhibitors such as flavopiridol may be clinically efficacious in relapsed Ewing sarcoma/PNET.

**Conclusion:**

Preclinical evidence exists that targeted therapeutics may be efficacious in the ESFT. IGF-1R antagonists have demonstrated efficacy in phase I/II clinical trials, although predicting responses remains a challenge. The future treatment of Ewing sarcoma/PNET is likely to be improved by these scientific advances.

## Introduction

Ewing sarcoma/PNET is a high grade malignancy in which approximately 75% of cases are localised at diagnosis, and 25% are initially metastatic [1-3]. The Surveillance Epidemiology and End Results (SEER) program reported an annual incidence rate of 2.93 cases/1,000,000 in the interval from 1973 to 2004 [3]. This low incidence has impaired the ability of clinicians to conduct prospective randomised controlled trials as frequently as is desirable. The general treatment paradigm for ESFT is chemotherapy with intercalated loco regional management with surgery with or without radiation treatment for patients with localized disease. The current overall disease free survival rate for metastatic disease is 25% and residual or recurrent Ewing sarcoma/PNET has a 10% overall survival rate. The Childhood Cancer Survivor Study issued a report in 2009 on late recurrence in paediatric cancers on a retrospective cohort of 12,795 survivors that had not recurred in the first 5 years post diagnosis. The greatest risk factor for late recurrence on multivariate analysis was a diagnosis of Ewing sarcoma/PNET or CNS tumour (astrocytoma), with adjusted rate ratios of 1.7 and 4.5 respectively. In the case of Ewing sarcoma/PNET, the cumulative incidence of late recurrence at 10 years was 9.4%, rising to 13% at 20 years [4]. For long-term survivors of childhood Ewing sarcoma/PNET (defined as patients that survived ≥ 5 years from diagnosis), the overall cumulative mortality of Ewing Sarcoma/PNET survivors was 25% when followed 25 years post diagnosis. Disease recurrence/progression accounted for 60.3% of deaths. Subsequent malignant neoplasms occur in 9% of survivors, and the risk of second cancers (particularly thyroid cancer, sarcoma and breast cancers) was increased by exposure to radiotherapy. There was also an increased risk of chronic health conditions (70.7% of survivors versus 33.7% of siblings) and infertility (the relative rate of pregnancy in survivors versus siblings was 0.65) [5]. There is an urgent need to improve cure rates for localized, metastatic and recurrent disease, while concurrently decreasing treatment related morbidity. Emergent targeted therapeutics offer many exciting possibilities in this disease and this publication concerns new molecular treatments for Ewing sarcoma/PNET tumours and evolving treatment paradigms that include targeted therapeutics. The field of improving treatment outcomes for patients with Ewing sarcoma/PNET by molecular therapeutics is hindered by the low frequency of Ewing sarcoma/PNET, the age demographics and technical obstacles such as therapeutics based on siRNA and cDNA oligonucleotides having drug delivery and degradation problems. Many of these problems potentially can be surmounted by increased collaboration between preclinical researchers and physicians caring for patients with Ewing sarcoma/PNET tumours.

## Ewing sarcoma/PNET tumours: an overview

Ewing sarcoma, peripheral primitive neuroectodermal tumours and Askin tumour of the chest wall belong to the Ewing sarcoma/PNET category of tumours. Although Ewing sarcoma/PNET tumours frequently are of osseous origin, 10% of cases of Ewing sarcoma/PNET tumours arise in extra skeletal soft tissues. It may arise from bone generating mesoderm however it does express neuroectodermal proteins. An emergent consensus favours it to be mesodermally derived [6]. Studies have found that inhibition of EWS-FLI expression in patient derived Ewing sarcoma/PNET cells lines causes these cells to adopt a mesenchymal stem cell phenotype [7,8]. There is a need for improving diagnostic tests to identify Ewing sarcoma. Many of the clinical, morphological and immunophenotypic characteristics of Ewing/PNET tumours are shared by other diseases such as small cell osteosarcoma and mesenchymal chondrosarcoma. Finding EWSR1 translocation can be very useful for deciding upon therapeutic management but an expansion in molecular disease identifiers is required particularly when one considers the combinatorial diversity among chromosomal breakpoints in Ewing sarcoma/PNET tumours.

Ewing sarcoma/PNET affects children's, adolescents and young adults with most cases occurring in the second and third decades of life. The median age at diagnosis ranges from 13 to 19 years in studies not restricted to paediatric recruitment. Interestingly adolescents and young adults (AYA) fare more poorly than children diagnosed with this disease, and is a subject for research. A 2003 study of standardized chemotherapeutic regimens in Ewing sarcoma/PNET had a 5 year survival rate for children less than 10 years of age of 70% compared with 60% for 10 to 17 year olds and 44% for 18 years and older. The reasons for poorer survival in the AYA demographic are complex including biologic heterogeneity, physician experience in differing treatment centres and stagnant improvements in the overall AYA cancer survival statistics. It also is the case that adults tend to present with a higher frequency of metastatic disease and primary sites in a central or pelvic location all of which are associated with a more unfavourable prognosis.

The past decade has seen significant improvements in outcomes for patients with Ewing sarcoma/PNET [9]. The European Intergroup Cooperative Ewing's sarcoma study group evaluated prognostic factors in Ewing's tumour/PNET of bone in 975 patients with a median follow-up of 6.6 years. Metastasis at diagnosis conferred a 5 year relapse free survival (RFS) of 22% versus 55% for patients without initial metastasis (P < .0001). In the group with metastasis at diagnosis, multivariate analysis showed that site (axial or other) and age cohort (≥ 15 years versus < 15 years) had a significant effect on relapse free survival [10]. Other established prognostic factors outlined in the Clinical Recommendations devised by the European Society of Medical Oncology are tumour volume, serum lactate dehydrogenase, axial location and age greater than 15 years [11].

**Figure 1:**
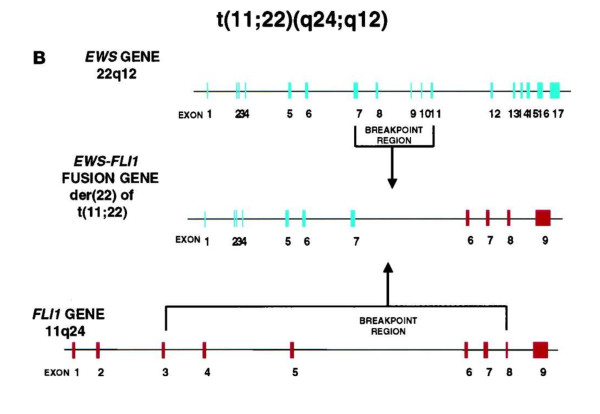
**Diagram of t(11;22)(q24;q12) and the EWS-FLI1 gene fusion**. Adapted from; Enrique de Alava, William L. Gerald, Biology of Sarcoma/Primitive Neuroectodermal Tumor Family, Journal of Clinical Oncology, Vol 18, Issue 1(January), 2000: 204.

Members of the Ewing sarcoma/PNET family of tumours are characterized by rearrangements involving the *EWS *gene on chromosome 22q12 and fusion partners from the *ETS *oncogene family, most frequently *FLI1 *on chromosome 11q24 (85%) as demonstrated in Figure 1, or *ERG *on chromosome 21q22 (10%). The EWS gene also has 3 other ETS family gene partners leading to the chimeric gene transcripts. The five most common gene rearrangements are detailed in table 1 however other rarer fusions have been described. Ninety-five percent of ESFT have fusion of the central exons of the EWSR1 gene (Ewing Sarcoma Breakpoint Region 1) to the central exons of an ETS gene family member and fusion occurs between the NH_2 _end of the EWS gene and the -COOH end of the ETS gene family partner. In Ewing sarcoma/PNET tumours it was previously considered that one particular rearrangement was specific for the malignancy and that no molecular shift occurs during progression. However a case has been described of a patient, in whom two separate Ewing sarcoma/PNET tumours arose after a 56 month remission interval, initially involving a EWS/ERG fusion transcript followed by a EWS/FLI1 fusion [12]. As Ewing sarcoma/PNET tumours have variable breakpoint locations in the involved genes, there is heterogeneity in fusion RNA and protein architecture. The effect of EWS-ETS fusion type on disease progression in Ewing sarcoma/PNET tumours was evaluated prospectively from the co-operative Euro-E.W.I.N.G. 99 trial [13]. Variants in the genomic breakpoint location lead to the transcription of varying RNA's. In most cases rearrangements occur with EWS intron 7 or 8: FLI1 introns 5 or 4 leading to either fusion of EWS intron 7 to Fli1 exon 6 (type I fusion [EF1]) documented in 51% of cases or exon 5 (type 2 fusion [EF2]) recorded in 27% of cases.

**Table 1:** Common Ewing sarcoma/PNET family of tumours chimeric fusion gene translocations: Most frequently implicated chimeric fusion gene translocations.

Genes	Translocation
EWS FLI1	t(11;22)(q24;q12)

EWS ERG	t(21;22)(q22;q12)

EWS ETV1	t(7;22)(p22;q12)

EWS E1AF	t(17;22)(q12;q12)

EWS FEV	t(2;22)(q33;q12) *

*Other ETS family partners: ETV4, PATZ1 (~5%), FUS-ERG (< 1%)

(Primer of the Molecular Biology of Cancer. Cancer Principles and Practice. Chapter 26; Sarcomas, p 334 Table 26.1).

This prospective study found no correlation with fusion type and prognosis. This refuted two previous smaller retrospective studies that suggested a correlation between fusion type and Ewing sarcoma/PNET prognosis [14,15]. Overall, current evidence does not support the use of translocation type for prognostication in Ewing sarcoma/PNET. This finding is also substantiated by a Children's Oncology Group prospective study published in 2010 [16]. In that study the association of type 1 EWS-FLI1 (fusion between exons 7 of EWS and 6 of FLI1) and non type 1 transcript translocations were correlated with disease characteristics, event free survival and overall survival. It was found that 89% of identified transcripts were EWS-FLI1 and of that cohort 58.8% were type 1. The event free survival at 5 years and overall survival did not show a clinical disadvantage to non-type 1 EWS-FLI1 fusions as had previously been suggested by retrospective studies. The analysis was restricted to patients with localised tumours that were diagnosed subsequent to 1994 and treated with COG protocols. It was postulated that advances in treatment in particular the routine inclusion of IE into Ewing sarcoma/PNET protocols eliminated the prognostic disadvantage of non-type 1 fusions.

## Chemotherapy in Ewing sarcoma/PNET

### Initial treatment

An overview of current cytotoxic management options in Ewing sarcoma/PNET is important as most emergent molecularly targeted therapeutics probably will be incorporated into existing treatment paradigms. The Children's Cancer Group and the Paediatric Oncology Group initiated a trial (National Cancer Institute, protocol INT-0091) on patients 30 years old or younger with Ewing sarcoma, primitive neuroectodermal tumour of bone, or primitive sarcoma of bone [17]. The study included patients with both localised and metastatic disease and the findings were published in 2003. Patients were randomly assigned to receive standard chemotherapy with doxorubicin, vincristine, cyclophosphamide, and dactinomycin or experimental therapy with these four drugs alternating with treatment with ifosfamide and etoposide. In patients without metastatic disease the 5 year EFS rate in the experimental arm was 69% versus 54% in the standard treatment cohort. Furthermore the overall 5 year survival rate for the experimental treatment was 72% versus 61% for the standard regimen with a relative risk of death of 1.6 for the standard treatment. The European Intergroup Cooperative Ewing Sarcoma Study-92 (EICESS-92) published in 2008, investigated whether cyclophosphamide has a similar efficacy to ifosfamide in standard risk patients and whether the addition of etoposide improves survival in high risk patients [18]. Cyclophosphamide appears to have a similar effect on event free survival and overall survival as ifosfamide, but also increased toxicity. The 3 year EFS difference observed was 6.8%, and there was an EFS hazard ratio of 0.80 for localised disease and 0.96 for metastatic disease favouring the etoposide containing regimen. This was consistent with the finding of the National Cancer Institute/Intergroup Ewing Tumour Group trial INT-0091. These studies therefore suggest that the addition of ifosfamide and etoposide to a standard regimen does not affect the outcome for patients with metastatic disease.

Dose intensification has been studied. A Children's Oncology Group study that was published in abstract form compared vincristine, doxorubicin and cyclophosphamide alternating with ifosfamide and etoposide treatment every 2 weeks with that of treatment every 3 weeks. The interval compression group had an improved event free survival at 3 years (76% vs. 65% 3, P = 0.028 [19]. The Children's Oncology Group also in 2009 reported a study National Cancer Institute protocol INT-0154 (Children's Cancer Group 7942/Pediatric Oncology Group 9354) which opened to institution between dates in 1995 and 1998. In this study the dose intensification of alkylating agents did not improve EFS and overall survival for patients with Ewing sarcoma/PNET [20]. Whether dose intensification may benefit a subgroup at particularly high risk remains an area of active research. Outcomes are significantly worse for patients with metastatic disease at diagnosis, prompting testing of dose-intensified regimens in this setting. The Euro-EWING 99 R3 study published in 2010 consisted of a single arm study of 281 patients with high-risk disease that received six cycles of vincristine, ifosfamide, doxorubicin, and etoposide, one cycle of vincristine, dactinomycin, and ifosfamide, local treatment (surgery and/or radiotherapy), and high-dose busulfan-melphalan followed by autologous stem-cell transplantation [19]. The estimated 3-year EFS from the start of high dose treatment/stem cell transplant was 45% for 46 children younger than 14 years. Overall this study has a 3-year EFS of 27% and an OS of 34%.

### Relapsed disease

Relapsed Ewing sarcoma/PNET is generally regarded as an incurable disease. A 2005 study reported that 49% of patients achieved a partial or complete response to second-line treatment, with a median duration of response of 27 months [21]. The five-year OS for relapsed patients was 23%. Multivariate analysis found a reduced risk of death following response to second line treatment (relative risk; RR 0.14), if the relapse free interval was greater than 24 months (RR 0.29) and for those receiving high dose treatment (RR 0.26). Despite this, the role of high dose chemotherapy with autologous stem cell transplantation in relapsed disease is uncertain. A 1995 report from the EBMT solid tumour registry that evaluated the impact of megatherapy in a subgroup of children in second complete remission with high risk Ewings/PNET tumours found they had actuarial EFS of 32% at 5 years [22]. While high dose therapy may be useful in some patients as consolidation treatment for patients with Ewing sarcoma/PNET in second complete remission, most reports are non-randomised studies of patients with responsive disease [23]. The role of high dose therapy remains a subject for research.

Newer cytotoxic agents have entered clinical use. Camptothecin derivatives are effective in Ewing sarcoma. While single agent treatment with irinotecan and topotecan has shown little efficacy in phase I and II studies of patients with refractory disease, combinations that have been tested include temozolomide with irinotecan, topotecan with cyclophosphamide and high dose ifosfamide [24]. Most studies are limited by small patient numbers and the tendency to combined evaluation of patients with refractory and relapsed disease leading to study population heterogeneity. The combination of irinotecan and temozolamide was retrospectively reported on a 20 patient series with recurrent/refractory disease from Memorial Sloan Kettering, NY. There were 5 complete and 7 partial responses (63% overall response rate). Median time to progression was 8.3 months for patients with recurrent/progressive ES and for the subset with recurrent ES it was 16.2 months [25]. In another study the combination of cyclophosphamide and topotecan for patients with refractory or relapsed Ewing tumours was reported on 54 patients by German investigators. 32.6% had a partial response and 26.5% had stable disease. Of 19 relapsed patients that attained a complete response, 52.6% maintained remission. After a median follow-up of 23.1 months 25.9% of patients were in continuous complete or partial remission [26]. Lastly, high-dose ifosfamide was described in a study in which 33 patients with relapsed or advanced disease and 4 patients that had progression during neoadjuvant treatment, with a 34% overall response rate [27]. Finally, trabectedin has been evaluated in metastatic Ewing sarcoma/PNET patients progressing after standard chemotherapy yielding a 10.3% partial response rate, while 13.7% of patients experienced stable disease. The 6 month progression-free survival was 25% [28].

## New treatment strategies in Ewing sarcoma/PNET tumours

The National Cancer Institute in the United States are conducting clinical trials in Ewing sarcoma as selected trial that in particular utilise targeted molecular therapeutic strategies are documented in table 2. There is an urgent need for novel therapeutic strategies, particularly for patients with advanced disease at diagnosis, or who relapse following definitive treatment. The following sections describe a range of targeted and other approaches which are currently under investigation.

**Table 2:** Selected National Cancer Institute, U.S.

Study Title	Phase protocol's ID	Sponsor/lead organisations	Important points	Primary objective
Phase I/II partially randomised study of Hedgehog antagonist GDC-0449 in combination with γ- Secretase Inhibitor RO4929097 in patients with advanced or metastatic sarcoma	Phase I/II MSKCC-10049 10-049,8406, NCT01154452	Sponsor: NCI Lead Organisation: Memorial Sloan-Kettering Cancer Centre	Age: 18 and over Advanced sarcoma (Phase Ib) Advanced, metastatic sarcoma (Phase II) Expected enrolment (EE)120	(i) To determine maximum tolerated dose of RO4929097 when combined with GDC-0449 in patients with advanced sarcoma (Phase Ib). (ii) To assess progression free survival (PFS) of patients with advanced, metastatic sarcoma treated with RO4929097 with or without GDC-0449.

Phase II study of AuroraA Kinase Inhibitor MLN8237 in paediatric patients with recurrent or refractory solid tumours or leukaemia	Phase II COG-ADVL0921 ADVL0921 NCT01154816	Sponsor: NCI Lead Organisation: Children's Oncology Group	Age: 1-21 years Entry criteria includes patients with histological confirmed Ewing sarcoma/peripheral primitive neuroectodermal tumour EE 190	Assess objective response rate in paediatric patients

Phase II study of Cixutumumab in patients with relapsed or refractory solid tumours Cixutumumab: A fully human IgG1 monoclonal antibody directed against IGF-1R *	Phase II COG-ADVL0821 ADVL0821 NCT00831844	Sponsor: NCI Lead Organisation: Children's Oncology Group	Age: 6 months-30 years Entry criteria includes patients with histological confirmed Ewing sarcoma/peripheral primitive neuroectodermal tumour EE140	(i) Assess response rate (ii) Toxicity Secondary outcome: Relationship between tumour expression of IGF-I, IGF-II, and IGF-IR and response*

Phase II study of MK8669 in patients with metastatic bone or soft-tissue sarcoma (8669-030) MK8669 is a small molecule mTOR inhibitor and rapamycin analogue. mTOR is a serine/threonine kinase located downstream of the PI3K/Akt signalling pathway	Phase II	Sponsor: Pharmaceutical/Industry Trial sites located in Japan	Age: 13 and over Exclusion criteria include CNS metastasis (unless successfully treated), prior treatment with rapamycin or rapamycin analogues, on-going therapeutic toxicity from anticancer treatment, intercurrent or historic disease that may potentially confound results	Evaluation of MK 8669 when administered as maintenance therapy to patients with metastatic bone or soft-tissue sarcoma in Japan

IMC-A12 in combination with temsirolimus (CCI-779) in patients with advanced cancers	Phase I 2007-0595 MDA-2007-0595 8109 NCT00678223 NCT00678769	Sponsor: NCI Lead organisation: M.D. Anderson Cancer Centre, University of Texas	Age: 16 and over 3-6 participants each dose level of IMC-A12 in combination with temsirolimus	(i) To establish the highest tolerable dose combination of IMC-A12 and temsirolimus that can be given to patients with advanced or metastatic cancer. (ii) Establish drug safety. Tolerability of dose scheduling Biomarker studies incorporated

A phase I study of NK cell infusion following allogeneic peripheral blood stem cell transplantation from related or matched unrelated donors in paediatric patients with solid tumours and leukaemia's	Phase I 110073 11-C-0073 NCT01287104	NCI	Age: 4-35 years Paediatric solid tumours (if ESFT must have ultra-high risk ESFT) Re: EWS; (i) If at initial diagnosis has bone or bone marrow metastasis may be enrolled if completed standard front line therapy (SFLT) that includes vincristine, cyclophosphamide, adriamycin, ifosfamide and etoposide (ii) Patients with recurrence of tumour at any site less than 1 year after completing SFLT or with subsequent recurrence any time after completing SFLT (iii) Patients with progressive or persistent disease while receiving standard front line chemotherapy	(i) To determine the safety, effectiveness, and immune system response of giving NK white blood cells to individuals who have received allogeneic HSCT. (ii) To identify possible treatment related side effects. Background: Based on laboratory evidence NK cells following allogeneic PBSCT may have a beneficial anti-cancer therapeutic effect

Studies of Temozolamide in combination with topotecan in refractory and relapsed paediatric solid tumours	Phase II CSET 2008/1378 NCT 00918320	Lead organization: Institut Gustave Roussy	Age: 6 months - 20 years. Eligibility criteria include: Confirmed paediatric solid tumour, relapsed/refractory tumours in which SFLT has failed, not more than 2 lines of prior chemotherapy, CT/MRI measurable disease	To determine that the combination of topotecan and temozolamide is effective in the treatment of relapsed and refractory solid neuroblastoma and other paediatric solid tumours.

PCI-24781 in combination with doxorubicin to treat sarcoma A broad-spectrum phenyl hydroxamic acid inhibitor of histone deacetylase (HDAC). Inhibits several isoforms of HDAC causing accumulation of highly acetylated histones and induction of chromatin remodelling. It also inhibits homologous recombination (HR) activity by inhibiting the expression of RAD51	Phase I, II	Lead organisation/Sponsor Massachusetts General Hospital	Age: 18 years and over PCI-24781 is considered to regulate genes involved in tumour growth.	To determine safety and maximum tolerated dose of PCI-24781 that can be safety given with doxorubicin (phase I) and the the safety and efficacy of PCI-24781 when combined with doxorubicin (phase II) in patients with advanced sarcomas.

Study in localised and disseminated Ewing sarcoma	Phase III	Lead organisation/sponsor: Medizinische Klinik und Poliklinik A-Universitaetsklinikum Muenster, Germany	Age: 4-50 years EWING 2008 is a joint protocol of European and North American Ewing sarcoma study groups. Open to all patients diagnosed with Ewing sarcomas, localised or metastatic, who are considered eligible for neoadjuvant chemotherapy.	Standard Risk R1: Randomised trial High Risk R2: Randomized trial Very High Risk R3: Randomized trial Refer to NCI website for full study details

Clinical trials in Ewing sarcoma www.cancer.gov Entered search terms: Ewing sarcoma/PNET. Search, restricted to treatment.

*ASCO Annual meeting Chicago, 2011. The combination of Cixutumumab and temsirolimus was well tolerated in a study of 17 Ewing sarcoma patients. Prolonger tumour reduction in excess of 6 months and one complete response was seen in 29% of heavily pre-treated patients with Ewing's sarcoma/PNET (Poster Discussion Session Board #23).

### EWS-FLI1 Gene Silencing

The t (11; 22) EWS-FLI1 translocation is seen in 85% of cases of Ewing sarcoma, while the t (11; 22) EWS-WT1 translocation is seen in Desmoplastic small round cell tumours. Indeed the same 5' portions of EWSR1 that are implicated in Ewing sarcoma are fused to WT1 in this distinct tumour entity. The NH2 terminus of the EWS gene fuses with the COOH end of the ETS gene partner in 95% of Ewing sarcoma family of tumours rendering it an attractive therapeutic target. EWS-FLI1 expression can be reduced by antisense oligonucleotides, antisense RNA (expressed from a vector) and small interfering RNA delivered by nanoparticles. EWS-FLI1 elimination through antisense cDNA and siRNA causes prolonged survival of ESFT xenograft bearing mice [21,29,30]. However, therapeutics based on siRNA and cDNA oligonucleotides have inherent bioavailability and administration problems, and are not readily applicable to the clinical setting. EWS-FLI1 furthermore lacks a fixed structure and the EWS-FLI1 protein product lacks enzymatic activity and is not associated with constitutive activation of a tyrosine kinase. Therefore designing therapeutics that target EWS-FLI1 protein interactions is more therapeutically feasible rather than targeting the EWS-FLI1 protein itself. For example, RNA helicase A (RHA) binds to EWS-FLI1 as part of the EWS-FLI1 transcriptional complex that includes RNA polymerase II, cyclic AMP response element-binding protein and RHA. Small molecule blockade with YK-4-279 of the oncogenic protein EWS-FLI1 interaction with RNA helicase A inhibits growth of Ewing sarcoma by inducing apoptosis and may also regulate the cell cycle protein, cyclin D1 [31,32]. One study combining EWS-FLI-1 antisense oligonucleotide and rapamycin efficiently induced the apoptotic death of EWS cells in culture through a caspase-dependent apoptotic process that involved restoration of the TGF β-induced pro-apoptotic pathway in vivo [33].

An alternative strategy involves targeting downstream signalling molecules. Researchers at the University of Alabama found that EWS-FLI1 induced manic fringe (MNFG) renders NIH3T3 cells tumorigenic [34]. MNFG is a member of the Fringe gene family required for establishing the dorsoventral wing boundary in Drosophila wing development, and plays a key role in the Notch pathway. Conceptually, it is plausible that γ-secretase inhibitors targeting the Notch pathway may have clinical efficacy in Ewing sarcoma/PNET. A Canadian study found that Ewing sarcomas express Notch receptors, ligands and HES1 a target gene of the Notch pathway [35]. Furthermore, inhibition of Notch signalling induced neural differentiation in Ewing sarcoma cell lines however inhibition of Notch was associated with only a small change in tumour growth potential.

A separate study from INSERM France and UCLA combined siRNA technique with microarray technology to find genes regulated by EWS/FLI1 [36]. The authors found two functional classes of regulated genes. The first group were extracellular matrix components including collagen and lysyl-oxidase enzymes. The second functional cluster included genes regulating signal transduction pathways including negative regulators of IGF-1, STAT, Wnt and MAPK. This study particularly found evidence that the constitutive activation of the IGF-1 pathway in Ewing sarcoma was partly attributable to transcriptional repression of the IGFBP-3 promoter. Ewing sarcoma IGFBP3 based therapeutics may partly obviate therapeutic resistance to IGF-1R antagonists, for instance the overall response rate of only 12.4% seen in the phase I/II study involving figitumumab [37-39]. Overall repression of IGFBP-3 appears to be important in the development of Ewing sarcoma/PNET and EWS-FLI1 can bind the IGFBP-3 promoter in-vitro and in-vivo and repress its activity [34,40].

### IGF-1R antagonists

The insulin-like growth factor 1 receptor (IGF-1R) is a transmembrane receptor with tyrosine kinase activity when it binds its ligands IGF-I and IGF-II. After ligand binding the phosphatidylinositol 3' kinase, RAS-MAPK and JAK-STAT (Janus kinase and signal transducers and activators of transcription) pathways are activated causing cellular proliferation and survival [41]. All Ewing sarcoma/PNET cells express IGF-1R and are stimulated by IGF-1 in autocrine loops [42]. IGF-1 also is stored in the bone matrix and is potentially liberated by tumour induced osteolysis, a paracrine effect [43]. IGF-1R has no described mutations, is occasionally amplified but is frequently over expressed. Epidemiologically it is notable that ESFT usually occur in the 2nd and 3rd decades of life with a peak incidence at age 15 when growth hormone mediated IGF-1 production would be expected to be maximal. Experimentally, the IGF-1R pathway is necessary for malignant transformation when the cells are transfected with EWS/FLI1 cDNA [44]. In Ewing sarcoma; it appears that increased autocrine stimulation may be due to increased IGF1 ligand and IGF-1R over-expression, as well as repression of of the inhibitory protein, IGFBP3 [45,46].

Therapeutics directed against IGF1R include either antibodies (for example, IgG1: R1507 and AMG479; IgG2: figitumumab) or else small molecule tyrosine kinase inhibitors. Striking clinical responses for most IGF-1R inhibitors in Ewing sarcoma patients have been documented [40,45]. A phase I expansion cohort study of figutumumab in 29 patients with Ewing sarcoma and other sarcomas of whom 16 had Ewing sarcoma found two patient's (both of whom had Ewing sarcoma) that, had objective responses (one complete response, one partial response). Eight patients had disease stabilization of whom 6 had Ewing sarcoma/PNET lasting 4 to 16 months [47]. In a separate phase I/II study of figitumumab in patients with refractory Ewing sarcoma/PNET and other sarcomas, 16 patients with Ewing were entered onto the phase 1 portion and no dose limiting toxicities attributable to figitumumab emerged at the prescribed dose escalated cohorts of 20 or 30 mg/kg IV q28d. Of 106 patients that were evaluable in the phase II stage of the trial, 15 had a partial response and 25 had stable disease. Median PFS and OS were 1.9 and 8.9 months respectively. Patients with pre-treatment IGF-1 levels greater than 110 ng/ml (n = 79) had a median overall survival of 10.5 months whereas those with baseline IGF-1 levels less than 110 ng/ml (n = 19) had a median overall survival of 4.5 months with an estimated OS hazard ratio between the two populations of 0.354. This preliminary data set suggests that figitumumab may be efficacious in treating Ewing sarcoma/PNET [39]. A phase I study of the fully human monoclonal antibody AMG479 was published in 2008 [34]. That study established that AMG 479 can be safely administered at a dose of 20 mg/kg IV Q2W. Of 53 enrolled patients, 12 had a diagnosis of Ewing/PNET. Despite being primarily a pharmacodynamic and pharmacokinetic study it is notable that one patient that had previously received 5 courses of chemotherapy with a histologic diagnosis of Ewing/PNET had a rapid response to AMG 479 with a partial response on positron emission tomography-CT on day 8. A complete response (CR) occurred by day 57. The patient remains in sustained CR at 30 months. Molecular genetics found that the patient had the t (11; 22) (q24; q12) translocation and the EWS-FLI1 type 1 fusion product (exon 7/exon 6) and immunohistochemistry of a lung metastasis showed IGF-1R expression. A further patient with a EWS-FLI 1 type III fusion product (exon 10/exon 6) had a partial response but discontinued therapy. Ten additional Ewing/PNET patients were enrolled in the expansion phase but none experience a response.

It is plausible that inhibition of IGF-1R ameliorates the negative feedback of IGF-1 on growth factor secretion at the pituitary level. It has been shown that growth hormone negative feedback is mediated by somatomedin-C at the hypothalamic and pituitary levels [48]. Additionally observed toxicities included hyperglycaemia, hypothyroidism, grade 3 thrombocytopenia (n = 8/53) and grade 3 transaminitis (n = 1) as well as grade 1 and 2 toxicities. Hyperglycaemia is a commonly observed adverse effect from IGF-1R targeted therapeutics, and it was observed that despite the fact that the extracellular domain of IGF-1R and the insulin receptor (INSR) are approximately 50% identical AMG 479 did not bind or block insulin binding to the INSR in non-clinical studies [49,50].

A MD Anderson initiated phase I study of R1507, a monoclonal antibody to the insulin like growth factor receptor-1 (IGF1R), was conducted on 37 patients with advanced solid tumours. Nine of the patients entered had a diagnosis of Ewing sarcoma. Efficacy results were not the main study end-points however 2 patients with Ewing sarcoma had durable partial responses of 11.5 and > 26 months each and 2 had stable disease [51]. In a phase II trial of 125 patients with recurrent or refractory Ewing sarcoma/PNET treated with R1507, 18 patients had a CR or PR (14.4%). Twelve patients responded for at least 6 weeks with a median duration of response of 25 weeks. Median survival was 7.6 months [52]. It also should be mentioned that inhibition of the IGF-1R receptor pathway by a small molecule IGF-1R antagonist (ADW742) was examined alone or combined with imatinib, doxorubicin and vincristine in Ewing tumour/PNET cell lines. Treatment reduced cellular proliferation, induced G1 arrest and apoptosis as well as leading to down regulation of IGF-1R, AKT, and mTOR phosphorylation [53]. 113 patients were treated in a multi-centre phase II trial of R1507 in patients with recurrent or refractory Ewing sarcoma/PNET. Median age was 25 with 57% of cases having a primary bone tumour and 43% an extra skeletal primary site. Overall complete/partial response rate was 10%. Median duration of response was 29 weeks and median survival was 7.6 months. Of 11 responses observed 10 were in patients that had presented with primary osseous tumours [54].

In addition to monoclonal antibody targeting of IGF-1R and small molecule tyrosine kinase inhibitors a third method to treat Ewing sarcoma based on IGF-1R involves increasing circulating insulin-like growth factor binding protein 3 (IGFBP-3) levels. Investigators at INSERM Paris found that IGFBP-3 was strongly induced upon treating Ewing sarcoma/PNET cells with EWS/FLI-1-specific small interfering RNAs and IGFBP-3 has been considered to be a potential anticancer molecule in Ewing sarcoma [36]. Exogenous IGFBP-3 inhibited EWS growth in monolayers and anchorage-independent conditions [55,56]. Insulin like growth factor binding protein 3 is a potential anticancer therapeutic in Ewing sarcoma. A low level of IGFBP-3 was observed in a panel of cell lines or clinical samples indicating constitutive activation of the IGF-1 pathway in Ewing sarcoma. It was observed that the mechanism of action of IGFBP-3 was considered to be essentially attributable to IGF-1 sequestration. Systemic administration of IGFBP-3 may be associated with putative toxicities like insulin resistance and osteoporosis. Systemic toxicities may be partly obviated by local administration, and as 90% of Ewing sarcoma/PNET as skeletal and a third of Ewing sarcoma/PNET metastasis are in a skeletal location this is therapeutically advantageous were IGFBP-3 to be used therapeutically.

Based on accumulated data from phase I and II clinical trials up to 2011, only approximately 10% of cases of Ewing sarcoma/PNET respond to IGF-1R directed therapeutics. Mechanisms of drug resistance and predictors of medication sensitivity need to be refined for IGF-1R directed therapeutics in this disease. Studies have also evaluated putative IGF-1R downstream pathways in Ewing sarcoma cells, and showed constitutive activation of Erk1/2 and Akt important mediators of the MAPK and PI3-K pathways [57]. The authors found that both the MEK/MAPK inhibitors (PD98059 and U0126) and PI3K inhibitor (LY294002) decrease Ewing sarcoma/PNET cell survival by induction of G1 blockade. MEK/MAP blockade also increases Ewing sarcoma/PNET cell sensitivity to doxorubicin and reduces the cells migratory ability. Another potential mechanism of resistance to IGF-1R targeted therapies is enhanced insulin receptor (IR) -A homodimer formation and IGF-2 production [58]. Resistance cells can switch from IGF-1/IGF-1R to IGF2/IR-A dependency to continue activation of AKT and ERK1/2, proliferation and metastasis (the IR can exist in two isoforms that differ at the carboxy terminus of the α subunit by 12 amino acids termed, IR-A and IR-B). Resistance to IGF-1R directed therapeutics is partly mediated by increased insulin receptor signalling. In cell line variants resistant to IGF-1R antagonism there was exclusive expression of the IR-A isoform and increased activation of ERK1/2, AKT and STAT-3 which correlate with the malignant phenotype. It appears treatment efficacy should be evaluated in relation to the malignant cellular IR-A:IGF-1R ratio. Tumours with a low IGF-1R:IR ratios are unlikely to have a good chance of benefiting from IGF-1R directed therapeutics. Overall the field of insulin like growth factors in physiologic processes and cancers of different subtypes including Ewing sarcoma/PNET is a fascinating, important and rapidly evolving topic. Excellent comprehensive reviews of the subject are available [59]. Finally there also is the theoretical potential for combining IGR1R inhibitors with other drugs such as mTOR inhibitors with the prospect of enhanced treatment efficacy.

### mTOR inhibition

A key component of the PI3K pathway is mTOR. Transformation by PI3K and AKT downstream of IGF-1 and PDGF is dependent on the phosphorylation and activation of the 40S ribosomal protein S6 kinase (p70^S6K^) and the phosphorylation of 4E-binding protein by mTOR. Different types of EWS/FLI1expresse differing levels of total and phosphorylated mTOR protein. The concept of treating malignancies with mTOR inhibitors arises from increased mTOR activity in inherited conditions such as tuberous sclerosis that has increased frequency of multi-organ hamartomas and renal cell cancers (approximately 2.5%). Tuberous sclerosis is caused by inactivating mutations of either of the TSC1 and TSC2 tumour suppressor genes that encode the cytoplasmic TSC1 (hamartin) and TSC2 (tuberin) proteins respectively. These proteins interact with and inhibit mTOR [60]. Investigators treating angiomyolipomas in tuberous sclerosis complex, lymphangioleiomyomatosis or PEComas with sirolimus found evidence of clinical activity [61,62]. The use of rapamycin as a cytostatic treatment has been evaluated in Ewing sarcoma/PNET cell lines with heterogeneous EWS/FLI1 fusion genes. Rapamycin was found to inhibit cell line proliferation by causing G1 phase arrest with concurrent down regulation of EWS/FLI1 protein and restoration of expression of TGF-β type 2 receptor [63]. TGF-β type 2 receptor is transcriptionally repressed in Ewing sarcoma cells, and exposure to rapamycin resulted in a marked increase in TGF-β receptor 2 mRNA in the cell lines tested with concomitant increased susceptibility to TGF-β inhibition of growth.

### Targeting the KIT oncoprotein

Expression of KIT protein, mutational status of the kit gene exons 9, 11, 13, and 17, exons 12 and 18 of the PDGFRA gene, and exon 12 of the PDGFRB gene was evaluated in 71 cases of Ewing sarcomas [64]. Twenty seven (38%) were immunohistochemically positive for KIT protein but activating mutations in c-kit were found in only 2 of the 71 cases (2.6%), within exon 9. No activating mutations in the PDGFRA and PDGFRB genes were found but PDGFRA exon 18 polymorphisms were documented. Evidently c-kit activating mutations were found not to be coincident with KIT protein expression in Ewing sarcoma/PNET [60]. Imatinib has clinical efficacy is another sarcoma dermatofibrosarcoma protuberans; that tumour usually has a t(17:22) translocation and a response to imatinib is seen in 50% of cases by targeting PDGFRA [65]. In a Children's Oncology Group phase II study of seventy patients with relapsed or refractory solid tumours treated with imatinib mesylate only one partial response was observed amongst 24 patients with Ewing sarcoma/PNET, with no responses observed in patients with osteosarcoma, neuroblastoma, or desmoplastic small round cell tumours [66].

### CD99-directed monoclonal antibody treatment

CD99 is a surface glycoprotein encoded by the MIC2 gene that is present on Ewing sarcoma/PNET cells, hematopoietic stem cells, testis, prostate, pancreatic cells and gastric mucosa as well as in lymphoblastic lymphoma, embryonal rhabdomyosarcoma and other soft tissue sarcomas [67]. The MIC2 gene is located on the pseudo autosomal region at the end of the short arms of the × and Y chromosomes [68,69]. Engagement of CD99 by a monoclonal antibody induces rapid tumour apoptosis through caspase independent mechanisms [70-72]. A study that targeted CD99 with the 0662 monoclonal antibody and doxorubicin in athymic mice found synergistic efficacy against tumour growth and metastasis with an improved survival [73]. CD99 is highly expressed on T lymphocytes, thymocytes and hematopoietic cells including CD34 hematopoietic progenitor cells. In vitro analysis of different blood cell lineages exposed to a therapeutically effective dose of CD99 directed monoclonal antibody for the Ewing cells found no important bone marrow toxicity, but raises theoretical concerns regarding the combination with cytotoxic agents.

### Immunotherapy

Investigators at St. Jude Children's Research Hospital and the University of Tennessee expanded natural killer (NK) cells ex-vivo over 2-3 weeks in culture using co-culture with K562-mb15-41BBL cells. Some EWS cell lines were found to be very sensitive to allogenic NK cells from healthy donors (median cytotoxicity 87.2% at 1:1 effector to target ratio). Blocking the NKG2D receptor (an activating receptor when engaged that trigger NK cytotoxicity) and DNAM-1 (a receptor expressed on resting and activated NK cells and other hematopoietic cells) attenuated cell killing. Ewing sarcoma/PNET expresses NKG2D and DNAM-1 ligands, however a poor correlation was observed between expression of these ligand and cytotoxicity. These data suggest that other mechanisms may be involved [74]. NK cells also demonstrated anti-EWS efficacy when infused into immunodeficient mice bearing EWS xenografts. Interleukin 2 was co-administered to prolong the survival of NK cells and multiple infusions were administered to optimize tumour directed cytotoxicity. The role of immunotherapies remains to be established in clinical studies.

### TRAIL (Tumour Necrosis Factor (TNF)-Related Apoptosis-Inducing Ligand)

The TNF receptor super family is a principle regulator of apoptosis and TNF- related apoptosis-inducing ligand (TRAIL) induces apoptosis in Ewing sarcoma/PNET cells. Seventy percent of these cells express the TRAIL death receptors DR4 and DR5s are very sensitive to TRAIL-induced caspase-8-mediated apoptosis [75]. Furthermore, in an in vivo model of Ewing sarcoma, a cationic lipid vector was used to deliver TRAIL resulting in growth inhibition. Although conceptually appealing, treatment efficacy may be ameliorated by cellular resistance due to TRAIL pathway signalling dysfunctions, such as imbalances in death/decoy receptors or c-FLIP up regulation [76,77]. Furthermore, TRAIL is expressed in a range of normal tissues potentially leading to nonspecific tumour targeting with concomitant treatment toxicities. Interferons can lead to the induction of TRAIL and preclinical models have demonstrated strong inhibition of Ewing tumour/PNET xenograft growth by the combination of human interferon-alpha or interferon-beta with ifosfamide [78-81].

### Histone Deacetylase (HDAC) Inhibitors

Inhibition of histone deacetylase affects acetylation of histones and other proteins, resulting in transcriptional de-repression of tumour suppressor genes. This latter effect was exemplified in a xenograft model in which the HDAC inhibitor MS-27-275 was able to induce and increase TGF-β receptor 2 mRNA, thereby restoring TGF- β signalling and inhibition of ES cell growth [82]. Other genes, including the histone methyltransferase enhancer of Zeste Homologue 2 (EZH2), mediate gene silencing and are dependent on histone deacetylase activity. EZH2 has been found to be important for EWS/FLI1 driven tumour growth and metastasis and down regulation of EZH2 inhibited tumour growth in in-vivo experiments [83]. HDAC inhibitors have also been shown to inhibit the growth of Ewing sarcoma/PNET cells in other reports [84,85]. However, drug resistant clones of Ewing sarcoma/ESFTEFT with strong resistance to HDAC inhibitors have been identified re-enforcing the imperative to continue the search for other targeted therapeutics that have activity in Ewing sarcoma/PNET in addition to HDAC inhibitors [86].

### NKX2.2: a Transcriptional Repressor in Ewing Sarcoma/PNET

NKX2.2 is a transcription factor that contains a transcriptional repressor domain, and is a EWS/FLI target. Using microarray analysis, NKX2.2 down-regulates genes that overlap with genes regulated by EWS/FLI1. The DNA repressor domain in NKX2.2 was necessary for oncogenesis in Ewing sarcoma/PNET, and is probably mediated by histone deacetylase complexes. This observation led to studies using the HDAC inhibitor, vorinostat. The transcription profile of A673 Ewing sarcoma cells following treatment with vorinostat was compared with cells in which NKX2.2 was knocked down, and showed considerable overlap (*P *< 0.001). Effectively vorinostat reverses the NKX2.2 mediated transcription signature [87]. These gene sets involve mesenchymal differentiation, neurogenesis (including the NKX2.2 gene), extracellular matrix and cytoskeleton genes and those involved in angiogenesis, and also overlap with genes discriminating between Ewing sarcoma/PNET cells and mesenchymal stem cells.

### Other Kinase Inhibitors

EWS-FLI1 also regulates levels of cell cycle regulators. In Ewing sarcoma/PNET cell lines, cyclin dependent kinase 4 (CDK4), cyclin D1, Rb, p27^KIP1 ^and c-Myc were consistently highly expressed, whereas p57^KIP2^, p15INK4B and p14ARF demonstrated undetectable or low expression levels. Inducible expression of EWS-FLI-1 led to up-regulation of c-Myc and marked down-regulation of p57^KIP2 ^[88]. p16^INK4a ^status predicted 2-year survival amongst patients with Ewing sarcoma/PNET (*P *< .001) [89]. Also cyclin-dependent kinase 2 (CDK2) was over-expressed in patients with poor outcomes in a microarray-based study [90]. Cyclin E is one of the genes regulated by the EWS-FLI1 protein. The Cyclin E/CDK2 complex phosphorylates and inactivates the key cell cycle regulator and tumour suppressor, the retinoblastoma protein. The small molecule pan-cyclin dependent kinase inhibitor, flavopiridol, efficiently suppresses cell growth in vitro and in vivo in ESFT cells [91]. Flavopiridol induces apoptosis and inhibit tumor growth in drug-resistant Ewing sarcoma/PNET cells, by inducing the cleavage of poly-ADP-ribose polymerase (PARP), which is required for homologous recombination of DNA base mismatches [92]. Flavopiridol has also been found to increase the ratio of the pro-apoptotic protein level (Bax) to the anti-apoptotic protein level (Bcl-2 and Bcl-X (L)) [93]. The paediatric preclinical testing program (PPTP) released results in 2010 of initial tests of the small molecule Aurora Kinase A (AURKA) inhibitor MLN8237 using the PPTP in vitro and in vivo panel. AURKA is important for centrosome maturation and spindle formation during mitosis. MLN8237 inhibited the growth of the majority of the in vivo panel cell lines that included five Ewing sarcoma/PNET lines. Of the Ewing sarcoma/PNET cell lines that were evaluated in xenograft models one had uniformly high activity for time to event, tumour growth delay and objective response (SK-NEP-1) and the remainder all had low or intermediate results [94].

## Conclusion

There are two major themes emerging from a review of molecular pathology and therapeutics in Ewing sarcoma/PNET. Firstly, risk-adapted conventional treatment may be important, particularly as a high proportion of cases of Ewing sarcoma/PNET occur in the paediatric age group where long term treatment related adverse sequelae have important survivorship implications. For example, in the Euro-EWING 99 Trial, age, tumour volume and extent of metastatic spread where found to be prognostic risk factors for patients with primary disseminated multifocal Ewing sarcoma/PNET treated with intensive multimodal therapy. Various molecular markers of prognosis have recently been identified, including BMI-1 expression as well as prognostic gene expression signatures, which may be used to guide therapy [95]. Unlike Hodgkin lymphoma, current cure rates for limited stage Ewing sarcoma/PNET with conventional therapy suggest it is unlikely that there will be an appetite for reduced dose intensity. The major opportunity will be to identify those at significantly increased risk, who might benefit from dose-intensification. Secondly, Ewing sarcoma/PNET is an area where the potential exists -- notwithstanding the different technical challenges -- for targeted therapeutics to impact upon advanced or relapsed disease [96]. There are natural challenges in development of novel therapeutic approaches in Ewing sarcoma/PNET. The low incidence rate probably presents the most significant obstacle to improving the outcome for patients diagnosed with Ewing sarcoma as it is a comparatively unattractive area economically for drug development. Nonetheless, the rapid accrual to the R1507 SARC Ewing sarcoma study showed that, with optimism and global collaboration, large numbers of patients can be accrued in short timeframes. Considering the demographics, high lethality, and defined molecular pathogenesis, targeted therapeutics of Ewing sarcoma/PNET family of tumour is an area of importance for collaborative international clinical trials.

## Competing interests

The authors declare that they have no competing interests.

## Authors' contributions

FCK conceived the manuscript aims, reviewed the literature and wrote the manuscript. DMT wrote and edited the manuscript. All authors read and approved the final manuscript.

## References

[B1] Rodriguez-Galindo C Spunt SL Pappo AS Treatment of Ewing sarcoma family of tumours: current status and outlook for the future Med Pediatr Oncol 2003 40 5 276 87 12652615 10.1002/mpo.10240

[B2] DeVita Hellman Rosenberg's Cancer Vincent T DeVita, Theodore S Lawrence Steven A Rosenberg, Ronald A DePinho, Robert A Weinberg Principles & Practice of Oncology Cancer: Principles & Practice

[B3] Esiashvili N Goodman M Marcus RB Jr Changes in incidence and survival of Ewing sarcoma patients over the past 3 decades: Surveillance Epidemiology and End Results data J Pediatr Hematol Oncol 2008 30 6 425 30 18525458 10.1097/MPH.0b013e31816e22f3

[B4] Wasilewski-Masker K Liu Q Yasui Y Leisenring W Meacham LR Hammond S Meadows AT Robison LL Mertens AC Late recurrence in pediatric cancer: a report from the Childhood Cancer Survivor Study J Natl Cancer Inst 2009 101 24 1709 20 19966206 10.1093/jnci/djp417 PMC2800799

[B5] Ginsberg JP Goodman P Leisenring W Ness KK Meyers PA Wolden SL Smith SM Stovall M Hammond S Robison LL Oeffinger KC Long-term survivors of childhood Ewing sarcoma: report from the childhood cancer survivor study J Natl Cancer Inst 2010 102 16 1272 83 Epub 2010 Jul 23 20656964 10.1093/jnci/djq278 PMC2948841

[B6] Suvà ML Riggi N Stehle JC Baumer K Tercier S Joseph JM Suvà D Clément V Provero P Cironi L Osterheld MC Guillou L Stamenkovic I Identification of cancer stem cells in Ewing sarcoma Cancer Res 2009 69 5 1776 81 Epub 2009 Feb 10 19208848 10.1158/0008-5472.CAN-08-2242

[B7] Tirode F Laud-Duval K Prieur A Delorme B Charbord P Delattre O Mesenchymal stem cell features of Ewing tumours Cancer Cell 2007 11 5 421 9 17482132 10.1016/j.ccr.2007.02.027

[B8] Pizzo Philip A David G Poplack Principles and Practice of Pediatric Oncology 6 ISBN 978-1-60547-682-7

[B9] Balamuth NJ Womer RB Ewing sarcoma Lancet Oncol 2010 11 2 184 92 20152770 10.1016/S1470-2045(09)70286-4

[B10] Cotterill SJ Ahrens S Paulussen M Jürgens HF Voûte PA Gadner H Craft AW Prognostic factors in Ewing's tumour of bone: analysis of 975 patients from the European Intergroup Cooperative Ewing's Sarcoma Study Group J Clin Oncol 2000 18 17 3108 14 10963639 10.1200/JCO.2000.18.17.3108

[B11] ESMO clinical recommendations Paulussen M Bielack S Jürgens H Casali PG On behalf of the ESMO Guidelines Working Group Ewing's sarcoma of the bone: ESMO Clinical Recommendations for diagnosis, treatment and follow-up Ann Oncol 2009 20 suppl 4 iv140 iv142 10.1093/annonc/mdp155 19454436

[B12] Bielack SS Kohler G A patient with two Ewing sarcomas with distinct EWS fusion transcripts NEJM 2004 350 p1364 13 March 25 10.1056/NEJMc032965 15044653

[B13] Le Deley MC Delattre O Schaefer KL Burchill SA Koehler G Hogendoorn PC Lion T Poremba C Marandet J Ballet S Pierron G Brownhill SC Nesslböck M Ranft A Dirksen U Oberlin O Lewis IJ Craft AW Jürgens H Kovar H Impact of EWS-ETS fusion type on disease progression in Ewing's sarcoma/peripheral primitive neuroectodermal tumour: prospective results from the cooperative Euro-E.W.I.N.G. 99 trial J Clin Oncol 2010 28 12 1982 8 Epub 2010 Mar 22 20308673 10.1200/JCO.2009.23.3585

[B14] Zoubek A Dockhorn-Dworniczak B Delattre O Christiansen H Niggli F Gatterer-Menz I Smith TL Jürgens H Gadner H Kovar H Does expression of different EWS chimeric transcripts define clinically distinct risk groups of Ewing tumour patients? J Clin Oncol 1996 14 4 1245 51 8648380 10.1200/JCO.1996.14.4.1245

[B15] de Alava E Kawai A Healey JH Fligman I Meyers PA Huvos AG Gerald WL Jhanwar SC Argani P Antonescu CR Pardo-Mindan FJ Ginsberg J Womer R Lawlor ER Wunder J Andrulis I Sorensen PH Barr FG Ladanyi M EWS-FLI1 fusion transcript structure is an independent determinant of prognosis in Ewing's sarcoma J Clin Oncol 1998 16 4 1248 55 9552022 10.1200/JCO.1998.16.4.1248

[B16] van Doorninck JA Ji L Schaub B Shimada H Wing MR Krailo MD Lessnick SL Marina N Triche TJ Sposto R Womer RB Lawlor ER Current treatment protocols have eliminated the prognostic advantage of type 1 fusions in Ewing sarcoma: a report from the Children's Oncology Group J Clin Oncol 2010 28 12 1989 94 Epub 2010 Mar 22 20308669 10.1200/JCO.2009.24.5845 PMC2860404

[B17] Grier HE Krailo MD Tarbell NJ Link MP Fryer CJ Pritchard DJ Gebhardt MC Dickman PS Perlman EJ Meyers PA Donaldson SS Moore S Rausen AR Vietti TJ Miser JS Addition of ifosfamide and etoposide to standard chemotherapy for Ewing sarcoma and primitive neuroectodermal tumour of bone N Engl J Med 2003 348 8 694 701 12594313 10.1056/NEJMoa020890

[B18] Paulussen M Craft AW Lewis I Hackshaw A Douglas C Dunst J Schuck A Winkelmann W Köhler G Poremba C Zoubek A Ladenstein R van den Berg H Hunold A Cassoni A Spooner D Grimer R Whelan J McTiernan A Jürgens H European Intergroup Cooperative Ewing Sarcoma Study-92. Results of the EICESS-92 Study: two randomized trials of Ewing sarcoma treatment--cyclophosphamide compared with ifosfamide in standard-risk patients and assessment of benefit of etoposide added to standard treatment in high-risk patients J Clin Oncol 2008 26 27 4385 93 18802150 10.1200/JCO.2008.16.5720

[B19] Ladenstein R Pötschger U Le Deley MC Whelan J Paulussen M Oberlin O van den Berg H Dirksen U Hjorth L Michon J Lewis I Craft A Jürgens H Primary disseminated multifocal Ewing sarcoma: results of the Euro-EWING 99 trial Clin Oncol 2010 28 20 3284 91 Epub 2010 Jun 14 10.1200/JCO.2009.22.9864 20547982

[B20] Granowetter L Womer R Devidas M Krailo M Wang C Bernstein M Marina N Leavey P Gebhardt M Healey J Shamberger RC Goorin A Miser J Meyer J Arndt CA Sailer S Marcus K Perlman E Dickman P Grier HE Dose-intensified compared with standard chemotherapy for nonmetastatic Ewing sarcoma family of tumors: a Children's Oncology Group Study J Clin Oncol 2009 27 15 2536 41 Epub 2009 Apr 6 19349548 10.1200/JCO.2008.19.1478 PMC2684856

[B21] Barker LM Pendergrass TW Sanders JE Hawkins DS Survival after recurrence of Ewing sarcoma family of tumours J Clin Oncol 2005 23 19 4354 62 Epub 2005 Mar 21 15781881 10.1200/JCO.2005.05.105

[B22] Ladenstein R Lasset C Pinkerton R Impact of megatherapy in children with high-risk Ewing's tumours in complete remission: A report from the EBMT Solid Tumour Registry Bone Marrow Transplant 1995 15 697 705 7670398

[B23] Marina N Meyers PA High-dose therapy and stem-cell rescue for Ewing's family of tumours in second remission J Clin Oncol 2005 23 19 4262 4 Epub 2005 Mar 21 15781877 10.1200/JCO.2005.12.915

[B24] Subbiah V Anderson P Lazar AJ Burdett E Raymond K Ludwig JA Ewing sarcoma: standard and experimental treatment options Curr Treat Options Oncol 2009 10 1-2 126 40 Epub 2009 Jun 17 19533369 10.1007/s11864-009-0104-6

[B25] Casey DA Wexler LH Merchant MS Chou AJ Merola PR Price AP Meyers PA Irinotecan and temozolomide for Ewing sarcoma: the Memorial Sloan-Kettering experience Pediatr Blood Cancer 2009 53 6 1029 34 19637327 10.1002/pbc.22206

[B26] Hunold A Weddeling N Paulussen M Ranft A Liebscher C Jürgens H Topotecan and cyclophosphamide in patients with refractory or relapsed Ewing tumors Pediatr Blood Cancer 2006 47 6 795 800 16411206 10.1002/pbc.20719

[B27] Ferrari S del Prever AB Palmerini E Staals E Berta M Balladelli A Picci P Fagioli F Bacci G Vanel D Response to high-dose ifosfamide in patients with advanced/recurrent Ewing sarcoma P ediatr Blood Cancer 2009 52 5 581 4 10.1002/pbc.21917 19142994

[B28] Dileo P Grosso F Casanova M Jimeno J Marsoni S Sanfilippo R Podda M Ferrari S Bertulli R Casali PG Trabectedin in metastatic Ewin's family tumours patients progressing after standard chemotherapy Journal of Clinical Oncology, 2007 ASCO Annual Meeting Proceedings 2007 25 18S (June 20 Supplement) 10040

[B29] Tanaka K Iwakuma T Harimaya K Sato H Iwamoto Y EWS-Fli1 antisense oligodeoxynucleotide inhibits proliferation of human Ewing sarcoma and primitive neuroectodermal tumour cells J Clin Invest 1997 99 2 239 47 9005992 10.1172/JCI119152 PMC507791

[B30] Hu-Lieskovan S Heidel JD Bartlett DW Davis ME Triche TJ Sequence-specific knockdown of EWS-FLI1 by targeted, nonviral delivery of small interfering RNA inhibits tumour growth in a murine model of metastatic Ewing sarcoma Cancer Res 2005 65 19 8984 92 16204072 10.1158/0008-5472.CAN-05-0565

[B31] Erkizan HV Kong Y Merchant M Schlottmann S Barber-Rotenberg JS Yuan L Abaan OD Chou TH Dakshanamurthy S Brown ML Uren A Toretsky JA A small molecule blocking oncogenic protein EWS-FLI1 interaction with RNA helicase A inhibits growth of Ewing sarcoma Nat Med 2009 15 7 750 6 Epub 2009 Jul 5 19584866 10.1038/nm.1983 PMC2777681

[B32] Erkizan HV Uversky VN Toretsky JA Oncogenic partnerships: EWS-FLI1 protein interactions initiate key pathways of Ewing sarcoma Clin Cancer Res 2010 16 16 4077 83 Epub 2010 Jun 14 20547696 10.1158/1078-0432.CCR-09-2261 PMC3682924

[B33] Mateo-Lozano S Gokhale PC Soldatenkov VA Dritschilo A Tirado OM Notario V Combined transcriptional and translational targeting of EWS/FLI-1 in Ewing sarcoma Clin Cancer Res 2006 12 22 6781 90 17121899 10.1158/1078-0432.CCR-06-0609

[B34] May WA Arvand A Thompson AD Braun BS Wright M Denny CT EWS/FLI1-induced manic fringe renders NIH 3T3 cells tumorigenic Nat Genet 1997 17 4 495 7 9398859 10.1038/ng1297-495

[B35] Baliko F Bright T Poon R Cohen B Egan SE Alman BA Inhibition of notch signaling induces neural differentiation in Ewing sarcoma Am J Pathol 2007 170 5 1686 94 17456774 10.2353/ajpath.2007.060971 PMC1854963

[B36] Prieur A Tirode F Cohen P Delattre O EWS/FLI-1 silencing and gene profiling of Ewing cells reveal downstream oncogenic pathways and a crucial role for repression of insulin-like growth factor binding protein 3 Mol Cell Biol 2004 24 16 7275 83 15282325 10.1128/MCB.24.16.7275-7283.2004 PMC479730

[B37] Rajah R Valentinis B Cohen P Insulin-like growth factor (IGF)-binding protein-3 induces apoptosis and mediates the effects of transforming growth factor-beta1 on programmed cell death through a p53- and IGF-independent mechanism J Biol Chem 1997 272 18 12181 8 9115291 10.1074/jbc.272.18.12181

[B38] Lee KW Cohen P Nuclear effects: unexpected intracellular actions of insulin-like growth factor binding protein-3 J Endocrinol 2002 175 1 33 40 Review 12379488 10.1677/joe.0.1750033

[B39] ESMO Conference, Milan October 2010 Proferred papers. 13440 Safety and efficacy results from a phase 1/2 study of the anti-IGF-IR antibody figitumab in patients with refractory Ewing and osteosarcomas Heribert Juergens, Muenster, Germany

[B40] Tolcher AW Sarantopoulos J Patnaik A Papadopoulos K Lin CC Rodon J Murphy B Roth B McCaffery I Gorski KS Kaiser B Zhu M Deng H Friberg G Puzanov I Phase I, pharmacokinetic, and pharmacodynamic study of AMG 479, a fully human monoclonal antibody to insulin-like growth factor receptor 1 J Clin Oncol 2009 27 34 5800 7 Epub 2009 Sep 28 19786654 10.1200/JCO.2009.23.6745

[B41] Choy E Digumarthy SR Koplin SA Case records of the Massachusetts General Hospital. Case 36-2009. A 23-year-old man with cough, hoarseness, and abnormalities on chest imaging N Engl J Med 2009 361 21 2080 7 19923580 10.1056/NEJMcpc0907804

[B42] Scotlandi K Benini S Sarti M Serra M Lollini PL Maurici D Picci P Manara MC Baldini N Insulin-like growth factor I receptor-mediated circuit in Ewing sarcoma/peripheral neuroectodermal tumour: a possible therapeutic target Cancer Res 1996 56 20 4570 4 8840962

[B43] Staal A Geertsma-Kleinekoort WM Van Den Bemd GJ Buurman CJ Birkenhäger JC Pols HA Van Leeuwen JP Regulation of osteocalcin production and bone resorption by 1,25-dihydroxyvitamin D3 in mouse long bones: interaction with the bone-derived growth factors TGF-beta and IGF-I J Bone Miner Res 1998 13 1 36 43 9443788 10.1359/jbmr.1998.13.1.36

[B44] Toretsky JA Kalebic T Blakesley V LeRoith D Helman LJ The insulin-like growth factor-I receptor is required for EWS/FLI-1 transformation of fibroblasts J Biol Chem 1997 272 49 30822 7 9388225 10.1074/jbc.272.49.30822

[B45] Is the indication of IGFR antobodies in advanced refractory sarcoma expanding? ESMO Meeting Milan, Italy 2010 Jean-Yves Blay, Lyon EORTC

[B46] Zhan S Shapiro DN Helman LJ Loss of imprinting of IGF2 in Ewing sarcoma Oncogene 1995 11 12 2503 7 8545106

[B47] Olmos D Postel-Vinay S Molife LR Okuno SH Schuetze SM Paccagnella ML Batzel GN Yin D Pritchard-Jones K Judson I Worden FP Gualberto A Scurr M de Bono JS Haluska P Safety, pharmacokinetics, and preliminary activity of the anti-IGF-1R antibody figitumumab (CP-751,871) in patients with sarcoma and Ewing sarcoma: a phase 1 expansion cohort study Lancet Oncology 2010 11 129 35 20036194 10.1016/S1470-2045(09)70354-7 PMC2941877

[B48] Berelowitz M Szabo M Frohman LA Firestone S Chu L Hintz RL Somatomedin-C mediates growth hormone negative feedback by effects on both the hypothalamus and the pituitary Science 1981 212 4500 1279 81 6262917 10.1126/science.6262917

[B49] Werner H Le Roith D The insulin-like growth factor-I receptor signaling pathways are important for tumorigenesis and inhibition of apoptosis Crit Rev Oncog 1997 8 1 71 92 9516087 10.1615/critrevoncog.v8.i1.40

[B50] Beltran PJ Mitchell P Chung YA Cajulis E Lu J Belmontes B Ho J Tsai MM Zhu M Vonderfecht S Baserga R Kendall R Radinsky R Calzone FJ AMG 479, a fully human anti-insulin-like growth factor receptor type I monoclonal antibody, inhibits the growth and survival of pancreatic carcinoma cells Mol Cancer Ther 2009 8 5 1095 105 Epub 2009 Apr 14 19366899 10.1158/1535-7163.MCT-08-1171

[B51] Kurzrock R Patnaik A Aisner J Warren T Leong S Benjamin R Eckhardt SG Eid JE Greig G Habben K McCarthy CD Gore L A phase I study of weekly R1507, a human monoclonal antibody insulin-like growth factor-I receptor antagonist, in patients with advanced solid tumours Clin Cancer Res 2010 16 8 2458 65 Epub 2010 Apr 6 20371689 10.1158/1078-0432.CCR-09-3220

[B52] Pappo AS Patel S Crowley J Reinke DK Staddon AP Kuenkele K Chawla SP Benjamin RS Helman LJ Baker LH Activity of R1507, a monoclonal antibody to the insulin-like growth factor-1 receptor (IGF1R), in patients (pts) with recurrent or refractory Ewing sarcoma family of tumours (ESFT): Results of a phase II SARC study. Abstract No: 10000 J Clin Oncol 2010 28 15s (suppl; abstr 10000) 10.1200/JCO.2010.34.0000 PMC3236654 22025149

[B53] Martins AS Mackintosh C Martín DH Campos M Hernández T Ordóñez JL de Alava E Insulin-like growth factor I receptor pathway inhibition by ADW742, alone or in combination with imatinib, doxorubicin, or vincristine, is a novel therapeutic approach in Ewing tumour Clin Cancer Res 2006 12 11 Pt 1 3532 40 16740780 10.1158/1078-0432.CCR-05-1778

[B54] Pappo AS Patel SR Crowley J Reinke DK Kuenkele KP Chawla SP Toner GC Maki RG Meyers PA Chugh R Ganjoo KN Schuetze SM Juergens H Leahy MG Geoerger B Benjamin RS Helman LJ Baker LH R1507, a Monoclonal Antibody to the Insulin-Like Growth Factor 1 Receptor in Patients With Recurrent or Refractory Ewing Sarcoma Family of Tumors: Results of a Phase II Sarcoma Alliance for Research through Collaboration Study Journal of Clinical Oncology 2011 10.1200/JCO.2010.34.0000 PMC3236654 22025149

[B55] de Alava E Panizo A Antonescu CR Huvos AG Pardo-Mindán FJ Barr FG Ladanyi M Association of EWS-FLI1 type 1 fusion with lower proliferative rate in Ewing sarcoma Am J Pathol 2000 156 3 849 55 10702401 10.1016/S0002-9440(10)64953-X PMC1876855

[B56] Benini S Zuntini M Manara MC Cohen P Nicoletti G Nanni P Oh Y Picci P Scotlandi K Insulin-like growth factor binding protein 3 as an anticancer molecule in Ewing sarcoma Int J Cancer 2006 119 5 1039 46 16570284 10.1002/ijc.21929

[B57] Benini S Manara MC Cerisano V Perdichizzi S Strammiello R Serra M Picci P Scotlandi K Contribution of MEK/MAPK and PI3-K signaling pathway to the malignant behavior of Ewing sarcoma cells: therapeutic prospects Int J Cancer 2004 108 3 358 66 14648701 10.1002/ijc.11576

[B58] Garofalo C Manara MC Nicoletti G Marino MT Lollini PL Astolfi A Pandini G López-Guerrero JA Schaefer KL Belfiore A Picci P Scotlandi K Efficacy of and resistance to anti-IGF-1R therapies in Ewing sarcoma is dependent on insulin receptor signaling Oncogene 2011 10.1038/onc.2010.640 21278796

[B59] Maki RG Small is beautiful: insulin-like growth factors and their role in growth, development, and cancer J Clin Oncol 2010 28 33 4985 95 Epub 2010 Oct 25 20975071 10.1200/JCO.2009.27.5040 PMC3039924

[B60] Paul E Thiele E Efficacy of sirolimus in treating tuberous sclerosis and lymphangioleiomyomatosis N Eng J Med 358 2 190 192 10.1056/NEJMe0707153 18184966

[B61] Bissler JJ McCormack FX Young LR Elwing JM Chuck G Leonard JM Schmithorst VJ Laor T Brody AS Bean J Salisbury S Franz DN Sirolimus for angiomyolipoma in tuberous sclerosis complex or lymphangioleiomyomatosis N Engl J Med 2008 358 2 140 51 18184959 10.1056/NEJMoa063564 PMC3398441

[B62] mTOR inhibitors From Lymphangioleiomyomatosis to Where? Paolo Casali, Milan, Italy, ESMO Conference 2010 Milan

[B63] Mateo-Lozano S Tirado OM Notario V Rapamycin induces the fusion-type independent downregulation of the EWS/FLI-1 proteins and inhibits Ewing sarcoma cell proliferation Oncogene 2003 22 58 9282 7 14681687 10.1038/sj.onc.1207081

[B64] Do I Araujo ES Kalil RK Bacchini P Bertoni F Unni KK Park YK Protein expression of KIT and gene mutation of c-kit and PDGFRs in Ewing sarcomas Pathol Res Pract 2007 203 3 127 34 Epub 2007 Feb 12 17298867 10.1016/j.prp.2006.12.005

[B65] Rutkowski P Van Glabbeke M Rankin CJ Ruka W Rubin BP Debiec-Rychter M Lazar A Gelderblom H Sciot R Lopez-Terrada D Hohenberger P van Oosterom AT Schuetze SM European Organisation for Research and Treatment of Cancer Soft Tissue/Bone Sarcoma Group; Southwest Oncology Group. Imatinib mesylate in advanced dermatofibrosarcoma protuberans: pooled analysis of two phase II clinical trials J Clin Oncol 2010 28 10 1772 9 Epub 2010 Mar 1 20194851 10.1200/JCO.2009.25.7899 PMC3040044

[B66] Bond M Bernstein ML Pappo A Schultz KR Krailo M Blaney SM Adamson PC A phase II study of imatinib mesylate in children with refractory or relapsed solid tumours: a Children's Oncology Group study Pediatr Blood Cancer 2008 50 2 254 8 17262795 10.1002/pbc.21132

[B67] Principles and Practice of Paediatric Oncology Pizzo PA, Poplack DG Ewing sarcoma family of tumours Chapet 32 5 P1007

[B68] Sohn HW Choi EY Kim SH Lee IS Chung DH Sung UA Hwang DH Cho SS Jun BH Jang JJ Chi JG Park SH Engagement of CD99 induces apoptosis through a calcineurin-independent pathway in Ewing sarcoma cells Am J Pathol 1998 153 6 1937 45 9846983 10.1016/S0002-9440(10)65707-0 PMC1866321

[B69] Scotlandi K Baldini N Cerisano V Manara MC Benini S Serra M Lollini PL Nanni P Nicoletti G Bernard G Bernard A Picci P CD99 engagement: an effective therapeutic strategy for Ewing tumours Cancer Res 2000 60 18 5134 42 11016640

[B70] Cerisano V Aalto Y Perdichizzi S Bernard G Manara MC Benini S Cenacchi G Preda P Lattanzi G Nagy B Knuutila S Colombo MP Bernard A Picci P Scotlandi K Molecular mechanisms of CD99-induced caspase-independent cell death and cell-cell adhesion in Ewing sarcoma cells: actin and zyxin as key intracellular mediators Oncogene 2004 23 33 5664 74 15184883 10.1038/sj.onc.1207741

[B71] Ambros IM Ambros PF Strehl S Kovar H Gadner H Salzer-Kuntschik M MIC2 is a specific marker for Ewing sarcoma and peripheral primitive neuroectodermal tumours. Evidence for a common histogenesis of Ewing sarcoma and peripheral primitive neuroectodermal tumours from MIC2 expression and specific chromosome aberration Cancer 1991 67 7 1886 93 1848471 10.1002/1097-0142(19910401)67:7<1886::aid-cncr2820670712>3.0.co;2-u

[B72] Alberto Pappo Carlos Rodriguez-Galindo, Fariba Navid, Joseph Khoury, Matthew Krasin Pediatric bone and soft tissue sarcoma Ewing sarcoma family of tumours Chapter 9 186

[B73] Scotlandi K Perdichizzi S Bernard G Nicoletti G Nanni P Lollini PL Curti A Manara MC Benini S Bernard A Picci P Targeting CD99 in association with doxorubicin: an effective combined treatment for Ewing sarcoma Eur J Cancer 2006 42 1 91 6 Epub 2005 Dec 2 16326096 10.1016/j.ejca.2005.09.015

[B74] Cho D Shook DR Shimasaki N Chang YH Fujisaki H Campana D Cytotoxicity of activated natural killer cells against pediatric solid tumours Clin Cancer Res 2010 16 15 3901 9 Epub 2010 Jun 11; Killing the Killer: Natural Killer Cells to treat Ewing sarcoma. Ahn YO, Weigel B, Verneris MR. Clin Cancer Res 2010 p 3819-3821; commentary 20542985 10.1158/1078-0432.CCR-10-0735 PMC3168562

[B75] Mitsiades N Poulaki V Mitsiades C Tsokos M Ewing sarcoma family tumours are sensitive to tumour necrosis factor-related apoptosis-inducing ligand and express death receptor 4 and death receptor 5 Cancer Res 2001 61 6 2704 12 11289151

[B76] Zh L Fang B Mechanisms of resistance to TRAIL-induced apoptosis in cancer Cancer Gene Ther 2005 12 228 37 15550937 10.1038/sj.cgt.7700792

[B77] Picarda G Lamoureux F Geffroy L Delepine P Montier T Laud K Tirode F Delattre O Heymann D Rédini F Preclinical evidence that use of TRAIL in Ewing sarcoma and osteosarcoma therapy inhibits tumour growth, prevents osteolysis, and increases animal survival Clin Cancer Res 2010 16 8 2363 74 Epub 2010 Apr 6 20371692 10.1158/1078-0432.CCR-09-1779

[B78] Alberto Pappo Pediatric bone and soft tissue sarcoma Chapter 9 P209

[B79] Fanger NA Maliszewski CR Schooley K Human dedritic cells mediate cellular apoptosis via tumour necrosis factor-related apoptosis-inducing ligand (TRAIL) J Exp Med 190 1155 1164 10523613 10.1084/jem.190.8.1155 PMC2195665

[B80] Sancéau J Poupon MF Delattre O Sastre-Garau X Wietzerbin J Strong inhibition of Ewing tumour xenograft growth by combination of human interferon-alpha or interferon-beta with ifosfamide Oncogene 2002 21 50 7700 9 12400012 10.1038/sj.onc.1205881

[B81] Abadie A Besançon F Wietzerbin J Type I interferon and TNFalpha cooperate with type II interferon for TRAIL induction and triggering of apoptosis in SK-N-MC EWING tumour cells Oncogene 2004 23 28 4911 20 15077162 10.1038/sj.onc.1207614

[B82] Jaboin J Wild J Hamidi H Khanna C Kim CJ Robey R Bates SE Thiele CJ MS-27-275, an inhibitor of histone deacetylase, has marked in vitro and in vivo antitumor activity against pediatric solid tumours Cancer Res 2002 62 21 6108 15 12414635

[B83] Richter GH Plehm S Fasan A Rössler S Unland R Bennani-Baiti IM Hotfilder M Löwel D von Luettichau I Mossbrugger I Quintanilla-Martinez L Kovar H Staege MS Müller-Tidow C Burdach S EZH2 is a mediator of EWS/FLI1 driven tumour growth and metastasis blocking endothelial and neuro-ectodermal differentiation Proc Natl Acad Sci USA 2009 106 13 5324 9 Epub 2009 Mar 16 19289832 10.1073/pnas.0810759106 PMC2656557

[B84] Nakatani F Tanaka K Sakimura R Matsumoto Y Matsunobu T Li X Hanada M Okada T Iwamoto Y Identification of p21WAF1/CIP1 as a direct target of EWS-Fli1 oncogenic fusion protein J Biol Chem 2003 278 17 15105 15 Epub 2003 Jan 30 12560328 10.1074/jbc.M211470200

[B85] Sakimura R Tanaka K Nakatani F Matsunobu T Li X Hanada M Okada T Nakamura T Matsumoto Y Iwamoto Y Antitumor effects of histone deacetylase inhibitor on Ewing family tumours Int J Cancer 2005 116 5 784 92 15849726 10.1002/ijc.21069

[B86] Okada T Tanaka K Nakatani F Sakimura R Matsunobu T Li X Hanada M Nakamura T Oda Y Tsuneyoshi M Iwamoto Y Involvement of P-glycoprotein and MRP1 in resistance to cyclic tetrapeptide subfamily of histone deacetylase inhibitors in the drug-resistant osteosarcoma and Ewing sarcoma cells Int J Cancer 2006 118 1 90 7 16049968 10.1002/ijc.21297

[B87] Owen LA Kowalewski AA Lessnick SL EWS/FLI mediates transcriptional repression via NKX2.2 during oncogenic transformation in Ewing sarcoma PLoS One 2008 3 4 e1965 18414662 10.1371/journal.pone.0001965 PMC2291578

[B88] Dauphinot L De Oliveira C Melot T Sevenet N Thomas V Weissman BE Delattre O Analysis of the expression of cell cycle regulators in Ewing cell lines: EWS-FLI-1 modulates p57KIP2and c-Myc expression Oncogene 2001 20 25 3258 65 11423975 10.1038/sj.onc.1204437

[B89] Honoki K Stojanovski E McEvoy M Fujii H Tsujiuchi T Kido A Takakura Y Attia J Prognostic significance of p16 INK4a alteration for Ewing sarcoma: a meta-analysis Cancer 2007 110 6 1351 60 17661343 10.1002/cncr.22908

[B90] Li X Tanaka K Nakatani F Matsunobu T Sakimura R Hanada M Okada T Nakamura T Iwamoto Y Transactivation of cyclin E gene by EWS-Fli1 and antitumor effects of cyclin dependent kinase inhibitor on Ewing family tumor cells Int J Cancer 2005 116 3 385 94 15818598 10.1002/ijc.21010

[B91] Li Y Tanaka K Li X Okada T Nakamura T Takasaki M Yamamoto S Oda Y Tsuneyoshi M Iwamoto Y Cyclin-dependent kinase inhibitor, flavopiridol, induces apoptosis and inhibits tumor growth in drug-resistant osteosarcoma and Ewing family tumor cells Int J Cancer 2007 121 6 1212 8 17520676 10.1002/ijc.22820

[B92] Slee EA Harte MT Kluck RM Wolf BB Casiano CA Newmeyer DD Wang HG Reed JC Nicholson DW Alnemri ES Green DR Martin SJ Ordering the cytochrome c-initiated caspase cascade: hierarchical activation of caspases-2, -3, -6, -7, -8, and -10 in a caspase-9-dependent manner J Cell Biol 1999 144 2 281 92 9922454 10.1083/jcb.144.2.281 PMC2132895

[B93] Boerner SA Tourne ME Kaufmann SH Bible KC Effect of P-glycoprotein on flavopiridol sensitivity. Selected active or intended National Cancer Institute clinical trials in Ewing sarcoma Br J Cancer 2001 84 10 1391 6 11355953 10.1054/bjoc.2000.1688 PMC2363628

[B94] Maris JM Morton CL Gorlick R Kolb EA Lock R Carol H Keir ST Reynolds CP Kang MH Wu J Smith MA Houghton PJ Initial testing of the aurora kinase A inhibitor MLN8237 by the Pediatric Preclinical Testing Program (PPTP) Pediatr Blood Cancer 2010 55 1 26 34 20108338 10.1002/pbc.22430 PMC2874079

[B95] Cooper A van Doorninck J Ji L Russell D Ladanyi M Shimada H Krailo M Womer RB Hsu JH Thomas D Triche TJ Sposto R Lawlor ER Ewing tumors that do not overexpress BMI-1 are a distinct molecular subclass with variant biology: a report from the Children's Oncology Group Clin Cancer Res 2011 17 1 56 66 Epub 2010 Nov 3 21047978 10.1158/1078-0432.CCR-10-1417 PMC3711406

[B96] Subbiah V Anderson P Targeted Therapy of Ewing Sarcoma Sarcoma. 2011 2011 686985 Epub 2010 Oct 31 10.1155/2011/686985 PMC2968715 21052545

